# Content and face validity of the Spanish version of the Sexual Self-Concept Inventory for early adolescent girls

**DOI:** 10.17533/udea.iee.v37n1e02

**Published:** 2019-01-06

**Authors:** Magda Liliana Villamizar Osorio, Elveny Laguado Jaimes

**Affiliations:** 1 Nurse, Ph.D. Professor, Universidad Cooperativa de Colombia. Bucaramanga (Colombia). email: magda.villamizar@campusucc.edu.co Universidad Cooperativa de Colombia Universidad Cooperativa de Colombia Colombia magda.villamizar@campusucc.edu.co; 2 Nurse; Magister. Assistant Professor, Cooperative University of Colombia (Universidad Cooperativa de Colombia). Bucaramanga (Colombia). email: elveny.laguado@campusucc.edu.co Universidad Cooperativa de Colombia Cooperative University of Colombia (Universidad Cooperativa de Colombia) Colombia elveny.laguado@campusucc.edu.co

**Keywords:** adolescent, female, semantics, comprehension, reproducibility of results, translations, self-concept., adolescente, femenino, semántica, comprensión, reproducibilidad de los resultados, traducciones, autoimagen., adolescente, feminino, semántica, compreensão, reprodutibilidade dos testes, traduções, autoimagen.

## Abstract

**Objective.:**

To determine the content and face validity of the Spanish version of the Sexual Self-Concept Inventory (O'Sullivan et al.) for early adolescent girls.

**Methods.:**

Instrument-based study in which the translation, back-translation and adaptation of the 34 items of the Sexual Self-Concept Inventory (SSCI) scale was performed. Five experts carried out the content and face validation; face validation included 35 girls from the municipality of Girón (Colombia).

**Results.:**

The version translated into Spanish has adequate content validity because all items exceeded the minimum CVI (0.58) value considered within an overall scale of 0.92. The face validity for the 35 early adolescent girls showed that 10 items of the instrument needed to be adjusted semantically and culturally.

**Conclusion.:**

The Spanish version of the scale is semantically and conceptually equivalent to the original scale and can be used in similar contexts to evaluate sexual self-concept in early adolescent girls.

## Resumen

**Objetivo.:**

Establecer la validez de contenido y facial de la versión en español del Inventario de Autoconcepto Sexual de O'Sullivan *et al*. para niñas en adolescencia temprana.

**Métodos.:**

Estudio de validación de una prueba en el que se realizó la traducción, retrotraducción y adaptación de los 34 ítems de la escala Sexual Self-Concept Inventory (SSCI). Se hizo validación de contenido y facial con 5 expertos y validación facial con 35 niñas del municipio de Girón (Colombia).

**Resultados.:**

La versión traducida al español tiene adecuada validez de contenido pues todos los ítems superan ICV mínimo de 0.58, siendo este valor para la escala total de 0.92. La validez facial en las 35 niñas mostró que 10 ítems del instrumento requerían ser ajustados semántica y culturalmente.

**Conclusión.:**

La escala en versión español posee equivalencia semántica y conceptual con la escala original y podría ser utilizada en contextos similares para evaluar el autoconcepto sexual en niñas en adolescencia temprana.

## Resumo

**Objetivo.:**

Estabelecer a validez de conteúdo e facial da versão em espanhol do Inventário de Auto-conceito Sexual de O'Sullivan et al. para meninas em adolescência precoce.

**Métodos.:**

Estudo de validação de um teste no qual se realizou a tradução, retro tradução e adaptação dos 34 itens da escala Sexual Self-Concept Inventory (SSCI). Se fez validação de conteúdo e facial com 5 especialistas e validação facial com 35 meninas do município de Girón (Colômbia).

**Resultados.:**

A versão traduzida ao espanhol tem adequada validez de conteúdo pois todos os itens superam ICV mínimo de 0.58, sendo este valor para a escala total de 0.92. A validez facial nas 35 meninas mostrou que 10 itens do instrumento tiveram que ser ajustados semântica e cultural.

**Conclusão.:**

A escala em versão espanhol possui equivalência semântica e conceitual com a escala original e poderia ser utilizada em contextos similares para avaliar o autoconceito sexual em meninas em adolescência precoce.

## Introduction

According to the World Health Organization (WHO),(1) adolescence is a complex transitional stage of life between childhood and adulthood (10 to 19 years of age) characterized by physical, psychological, biological, intellectual and social changes. It is subdivided into first or early adolescence-10 to 14 years of age-and second or late adolescence-15 to 19 years of age. Adolescence is not only a stage of vulnerability but also of opportunity; it is the time for development, wherein risks and vulnerabilities are faced, as well as for preparing to develop the entire potential within each adolescent.(2) Borges(2) states that in order to understand adolescents' behaviors and to exert influence over them, it is important to understand the environment surrounding adolescents and how this environment affects their development, behavior and social relationships. These environments include family, peer groups, school, work and leisure activities; these environments are simultaneously in a state of constant change. This conceptualization implies that interventions must consider these dimensions and include the adolescents themselves, without excluding the role of health professionals.

Adolescence is a stage of human development involving physical changes strongly marked by sexual maturity, that is, the development of secondary sexual characteristics. However, these characteristics do not depend on the roles, duties and rights intrinsic to this stage, thus providing one explanation for early initiation into sexual practices during adolescence. Young people are more vulnerable to sexually transmitted infections, mainly for behavioral reasons. Additionally, at this stage, adolescents develop emotions related to genital sexual maturity, due to greater interest in the opposite sex and in the same sex.(3) Also important to consider are the sexual cognitions of early adolescents as they relate to pubertal development. These cognitions are associated with changes in sexual expectations and roles, for which girls develop social meanings and feelings, as evidenced by how puberty transforms the manner in which the mother-daughter relationship is handled.(4) Likewise, it requires an understanding of adolescent girls’ sexual and reproductive health expectations(5-8) and the expression of sexuality through early adolescent girls’ behavior.(9) Sexual cognitions in early adolescent girls (negative sexual affect, sexual agency and sexual arousal) are present when they determine their sexual self-concepts.(4) The English version of the Sexual Self-Concept Inventory has allowed assess of this domain(9) by allowing the instrument’s measurements to precede sexual experiences.(10)

Thus, the first sexual experience of adolescents are related to desire, curiosity and, in some cases, pressure from their counterparts or friends who have had their first sexual relationship.(11) Therefore, health-prevention programs and the role of nursing are key to help early adolescent girls define their sexual self-concepts in order to improve their normative beliefs and encourage healthy sexual behaviors.(12,13) In addition, helping adolescent girls understand the meanings regarding the subjective and social processes during adolescence is important,(11) since there is lack of interventions related to early adolescent girls’ sexual health behavioral intentions and their sexual self-concepts.(12) In Colombia, no Spanish-language studies or instruments related to the sexual self-concept of early adolescent girls between 10 and 14 years of age were found. Likewise, the expression of the sexuality of adolescent girls through sexual self-concept has not been addressed. Therefore, supported by the presence of a validated English-version instrument, the study aimed to determine the content and face validity of the Spanish version of the Sexual Self-Concept Inventory of O'Sullivan *et al.*(4) for early adolescent girls, in order to carry out the transcultural adaptation of the instrument and apply it to the Colombian context.

## Methods

This research was based on a quantitative instrumental methodological design to determine content and face validity. The methodological aspects are described as follows.

*Selection of the instrument.* The Sexual Self-Concept Inventory (SSCI) was selected because it is an instrument that evaluates sexual self-concept in early adolescent girls. This instrument consists of three dimensions: sexual arousal, sexual agency and negative sexual affect. Its output offers a means to evaluate sexual behavior in adolescent girls and to assist the decision-making of nursing professionals with regard to risk. In addition, its results contribute to the development of research.(9) The SSCI comprises 34 items (sexual arousal = 17 items, sexual agency = 10 items and negative sexual affect = 7 items). It includes Likert-type response options on a scale of 1 to 6, with higher scores representing greater risk of sexual behavioral intention and expectation of sexual activity. The original version of the SSCI has demonstrated content validity, construct validity, and reliability. The percentage of explained variation is 40.2% for the 34 items. The total Cronbach's alpha for the scale is 0.91 (sexual arousal = 0.91, sexual agency = 0.76 and negative sexual affect = 0.67).(9)

*Process of translation and revision of official translations.* Three official translators were selected in order to generate a Spanish version closest to the original instrument in terms of construct, grammar and SSCI context. Once the translation was generated, experts in ​​early adolescence and sexual and reproductive health within the research group conducted a process to verify and review the Spanish version to choose the translation that best matched the context for Santander, Colombia.(14-16)

*Content validity.* Content was validated through the assessment of 5 experts (4 females, 1 male) with research and teaching experiences in sexual and reproductive health and adolescence, whose inclusion criteria included having a postgraduate degree in health (specialization, masters or doctorate) and 10 or more years of experience. The process was conducted according to the Lawshe index, modified by Tristán,(17) which measures ​​each item in three categories-essential, useful but not essential, and not necessary (for the overall scale)-in order to corroborate if each item adequately represented the sample of content.(18) Five experts were contacted personally and via email, each of whom conducted an individual assessment. Subsequently, the researchers consolidated the information for each item with their observations and made decisions, considering items that exceeded the minimum CVI value ≥ 0.58 as not needing to be modified.(17)

*Face validity.* We contacted 5 additional female experts in the research and teaching of sexual and reproductive health and adolescence, whose inclusion criteria included having a postgraduate degree in health (specialization, masters) and 10 or more years of experience. Our experts evaluated the 34 items of the instrument according to the following criteria: clarity (the type of language or easy-to-understand wording), precision (expression in a concise and exact language, leaving no doubts) and comprehension (understanding of what is meant by reading the item)(19). It was then decided that values ​​closest to 80% comprehensibility would be considered satisfactory.(20) Subsequently, the Kappa index was calculated to evaluate the index of inter-observer agreement;(21) values ​​between 0.61 and 0.80 were assumed to represent acceptable substantial agreement, and values of 0.81 or higher were assumed to represent superior acceptability agreement.(20) For face validity, 35 early adolescent girls were selected, among whom the instrument was assessed according to the comprehensibility criteria. The items’ comprehensibility was determined by the following percentages: equal to or greater than 85% = high comprehensibility; 80-85% = medium comprehensibility; and less than 80% = low comprehensibility. This study was carried out in the municipality of Girón (Santander, Colombia) in 2017. Subsequently, the information from the experts and the participants was consolidated separately, in order to perceive the clarity, precision and comprehension of the 34 items, which led to a final consensus on each item and consolidation of the consensus version. 

*Back translation.* This phase consisted of sending the final Spanish version of the instrument, adapted to the local context, to two official translators different from the initial translators to have it back-translated from Spanish to English. Subsequently, it was sent for verification and authorization of the changes made by the authors, who approved the Spanish version of the instrument.

*Ethical considerations*. Resolution 8430 of 1993 and Law 911 of 2004, chapter IV, articles 29, 30 and 34 of the Republic of Colombia were considered. This research was endorsed by the Ethics Committee of the Cooperative University of Colombia *(Universidad Cooperativa de Colombia)*. All phases included prior informed consent from both parents and assent of the girls prior to the explanation of the study. During the development of the study, no intervention or care for any adolescent girl due to emotional disturbance was required.

## Results

### Content validity

Table 1 shows the CVIs according to the expert assessments obtained for each item. Additionally, the table shows that the overall validity index of the 34 items was 0.92, a value considered acceptable. Consequent to these findings, all items were maintained.

**Table 1:** Content validity index according to the five experts, by dimension and item

Dimension	Item	Essential	Useful; non- essential	Not necessary	CVI
Sexual arousal	SE01	5	0	0	1
	SE02	4	1	0	0.6
	SE03	4	1	0	0.6
	SE04	4	1	0	0.6
	SE05	4	1	0	0.6
	SE06	4	1	0	0.6
	SE07	5	0	0	1
	SE08	5	0	0	1
	SE09	5	0	0	1
	SE10	5	0	0	1
	SE11	4	1	0	0.6
	SE12	5	0	0	1
	SE13	5	0	0	1
	SE14	4	1	0	0.6
	SE15	5	0	0	1
	SE16	4	1	0	0.6
	SE17	5	0	0	1
Sexual agency	SA01	5	0	0	1
	SA02	5	0	0	1
	SA03	5	0	0	1
	SA04	4	1	0	0.6
	SA05	5	0	0	1
	SA06	4	1	0	0.6
	SA07	4	1	0	0.6
	SA08	5	0	0	1
	SA09	5	0	0	1
	SA10	5	0	0	1
Negative sexual affect	NE01	5	0	0	1
	NE02	5	0	0	1
	NE03	5	0	0	1
	NE04	4	1	0	0.6
	NE05	4	1	0	0.6
	NE06	5	0	0	1
	NE07	5	0	0	1

### Face validity

Regarding the precision of the scale, the Kappa index of inter-observer agreement had a value of 0.9, which corresponds to almost perfect agreement. The experts clarified the questions with regard to concept and terminology; thus, it was necessary to review the items from the linguistic approach, with the help of an expert in Spanish language and literature.

**Table 2:** Inter-rater agreement of the five experts regarding the categories of clarity, comprehension and precision for the SSCI items

DIMENSION	ITEMS	CLARITY	COMPREHENSION	PRECISION
SEXUAL AROUSAL	SE01	0.6	0.4	1
	SE02	1	1	1
	SE03	1	0.6	1
	SE04	1	1	1
	SE05	1	1	1
	SE06	1	1	1
	SE07	1	1	1
	SE08	1	1	1
	SE09	0.4	0.6	1
	SE10	1	1	1
	SE11	1	1	1
	SE12	1	1	1
	SE13	1	0.6	1
	SE14	1	1	1
	SE15	1	1	1
	SE16	1	0.6	1
	SE17	1	1	1
SEXUAL AGENCY	SA01	1	0.6	0.6
	SA02	1	1	1
	SA03	1	0.6	0.6
	SA04	1	1	1
	SA05	1	1	1
	SA06	0.6	1	0.6
	SA07	1	1	1
	SA08	0.4	1	0.4
	SA09	1	1	1
	SA10	1	1	1
NEGATIVE SEXUAL AFFECT	NE01	1	1	1
	NE02	1	1	1
	NE03	1	1	1
	NE04	1	1	1
	NE05	1	1	1
	NE06	1	1	1
	NE07	1	1	1

Regarding face validity of the scale for the group of 35 early adolescent girls, the items’ degree of comprehensibility was determined according to the following percentages: equal to or greater than 85% (13 items) = high comprehensibility; 80-85% (6 items) = medium comprehensibility; and less than 80% (15 items) = low comprehensibility (see Table 3).

**Table 3:** Inter-rater agreement for the 35 early adolescent girls regarding the comprehension categories for the SSCI items

DIMENSION	ITEMS	%
SEXUAL AROUSAL	SE01	82.86
	SE02	85.71
	SE03	65.71
	SE04	57.14
	SE05	77.14
	SE06	80
	SE07	40
	SE08	91.43
	SE09	65.71
	SE10	80
	SE11	62.86
	SE12	82.86
	SE13	68.57
	SE14	74.29
	SE15	74.29
	SE16	85.71
	SE17	82.86
SEXUAL AGENCY	SA01	85.7
	SA02	94.3
	SA03	71.4
	SA04	91.4
	SA05	97.1
	SA06	65.7
	SA07	94.3
	SA08	74.3
	SA09	71.4
	SA10	74.3
NEGATIVE SEXUAL AFFECT	NE01	91.4
	NE02	71.4
	NE03	82.9
	NE04	85.7
	NE05	94.3
	NE06	94.3
	NE07	85.7

The results obtained from the adolescent girls show that 10 items of the instrument had values lower than 80; thus, these were discussed and adjusted from a semantic, conceptual and cultural approach, to be better understood by the early adolescent girls. Table 4 shows that after reviewing the 34 items, 76.5% were classified as semantically and conceptually equivalent to the original version, without needing to be modified, whereas 5.8% needed to be modified to achieve cultural equivalence.

**Table 4:** Classification of the equivalence of translations

ITEM	ORIGINAL VERSION	TRANSLATION	CONSENSUAL VERSION	OBSERVATIONS
SA010	If I have sex with a guy, I would worry that I could get my feelings really hurt.	Si tengo relaciones sexu- ales con un chico, me preocuparía que pudiera lastimar mis sentimientos.	Si tengo relaciones sexu- ales con un chico, me preocuparía que pudiera lastimar mis sentimientos.	Semantically and conceptually equivalent to the original version, without needing to be modified.
NE01	If I kiss a guy I don’t really know, I’m wor- ried of what people will think about me.	Si tengo relaciones sexu- ales con un chico, mis amigas quisieran saber todo acerca de ello.	Si tengo relaciones sexu- ales con un chico, mis amigas quisieran saber todo acerca de ello.	Semantically and conceptually equivalent to the original version, without needing to be modified.
NE02	Sex is nasty.	El sexo es desagradable.	El sexo es desagradable.	Semantically and conceptually equivalent to the original version, without needing to be modified.
NE03	Sex isn’t fun for girls my age.	El sexo no es divertido para las chicas de mi edad.	El sexo no es divertido para las chicas de mi edad.	Semantically and conceptually equivalent to the original version, without needing to be modified.
NE04	I would be scared to be really alone with a boy- friend.	Realmente me asustaría estar sola con un novio.	Realmente me asustaría estar sola con un novio.	Semantically and conceptually equivalent to the original version, without needing to be modified.
NEO5	Some girls have sex just to be accepted or popular.	Algunas chicas tienen sexo solo para ser aceptadas o populares.	Algunas chicas tienen sexo solo para ser aceptadas o populares.	Semantically and conceptually equivalent to the original version, without needing to be modified.
NE06	I think I am too young to have sex.	Creo que soy demasiado joven para tener sexo.	Creo que soy demasiado joven para tener sexo.	Semantically and Conceptually equivalent to the original version, without needing to be modified.
NE07	If I have sex, my friends will want to know all about it.	Si tengo relaciones sexu- ales con un chico, mis amigas quisieran saber todo acerca de ello.	Si tengo relaciones sexu- ales con un chico, mis amigas quisieran saber todo acerca de ello.	Semantically and conceptually equivalent to the original version, without needing to be modified.

## Discussion

Generating an instrument adapted to the Spanish language and to the Colombian culture of the municipality of Girón, Santander, becomes a starting point for measuring sexual self-concept in early adolescent girls, thereby strengthening aspects of interventions and research that allow this issue to be addressed and that contribute to the sexual and reproductive health of adolescent girls, which are essential in the progress of nursing. Sexual self-concept determines sexual behavioral intentions,(9) because it evaluates the individual’s own feelings and sexual actions, which implies a reflection and evaluation of the individual and is considered a predictor of sexual activity. By evaluating sexual self-concept, the early adolescent girls’ intentions to undertake health behaviors and prevent risk behaviors can be predicted.(12) This perspective is useful for nursing professionals in order to know how to address this issue.

Due to the scarcity of measurement instruments for this discipline, it was necessary to carry out the transcultural adaptation process. Regarding the instruments face validity, its clarity, comprehension, and precision were assessed through the participation of experts. The kappa index of inter-observer agreement characterizes values ​​between 0.61 and 0.80 as acceptable substantial agreement and of 0.81 and higher as agreement of superior acceptability; the study showed most items scored with a clarity value of 0.86, a precision value of 0.83 and a comprehension value of 0.89. Fleiss’ Kappa index showed substantial agreement in clarity and precision (0.75 and 0.72, respectively) and almost perfect agreement in comprehension (0.81).(22) In contrast, another study that used Fleiss’ Kappa index assumed values among examiners from 0.41 to 0.60 as acceptable agreement and values ​​greater than 0.61 as good or very good.(23) Regarding the face validity of the participants, values between 80 to 85% are considered to represent high comprehensibility, contrary to the study of validity and reliability of a professional care scale in its Spanish version, where a level of comprehensibility was set at greater than 95%, with an error of 8%, thus applying greater rigor in concluding adequate comprehensibility of the question.(24)

The content validity index obtained by the experts for the 34 items was 0.92; such value is considered acceptable, different from a study of validity and reliability of the Spanish version of the "Measurement of Self-Efficacy Perceived in Sleep Apnea" (SEMSA) instrument, in which 23 items reached a satisfactory level of acceptability and the remaining 4 items were subject to modification.(25) Compared to a study of validity and reliability of the Spanish version of the Technological Competency as Caring in Nursing instrument,(22) assessing each item under the criteria of Belonging and Relevance and qualifying each of them (per Denise Polit (26)) as 0 = Not pertinent/relevant, 1 = Slightly pertinent/relevant and 2 = Pertinent or relevant, the modified content validity index of Lawshe’s relevance criterion was 0.9, with high degree of agreement between the experts and meeting the relevance criterion of 0.9. This result was considered of great importance. Thus, a process of transcultural adaptation is necessary, with semantic adequacy to ensure clarity and comprehensibility by the subject of study. However, researchers must temper their efforts in terms of fidelity to the reproduction of content and interpretation of the items of the construct.(27)

The conclusion of this study is that the Spanish version of the SSCI is semantically and conceptually equivalent to the original scale and could be used in similar contexts to evaluate sexual self-concept in early adolescent girls. 

## References

[1] Organización Mundial de la Salud (OMS) (2010). Empoderamiento de mujeres adolescentes: un proceso clave para lograr los Objetivos de Desarrollo del Milenio.

[2] Borges AL (2007). Pressão social do grupo de pares na iniciação sexual de adolescentes. Rev. Esc. Enferm. USP.

[3] Chacón-Quesada T, Corrales-González D, Garbanzo-Núñez D, Gutiérrez-Yglesias JA, Hernández-Sandí A, Lobo-Araya A (2009). ITS Y SIDA en adolescentes: descripción, prevención y marco legal. Med. Leg Costa Rica..

[4] O’Sullivan LF, Meyer-Balhburg HF, Watkins BX (2000). Social cognitions associated with pubertal development in a sample of urban, low-income, African-American and Latina girls and mothers. J. Adolesc. Health.

[5] Coley RL, Lombardi CM, Lynch AD, Mahalik JR, Sims J (2013). Sexual partner accumulation from adolescence through early adulthood: The role of family, peer, and school social norms. J. Adolesc. Health.

[6] Manlove J, Wildsmith E, Ikramullah E, Terry-Humen E, Schelar E (2012). Family environments and the relationship context of first adolescent sex: Correlates of first sex in a casual versus steady relationship. Soc. Sci. Res.

[7] Markham CM, Peskin MF, Shegog R, Baumler ER, Addy RC, Thiel M (2014). Behavioral and psychosocial effects of two middle school sexual health education programs at tenth-grade follow-up. J. Adolesc. Health.

[8] Peres CA, Rutherford G, Borges G, Galano E, Hudes ES, Hearst N (2008). Family structure and adolescent sexual behavior in a poor area of São Paulo, Brazil. J. Adolesc. Health.

[9] O'Sullivan LF, Meyer‐Bahlburg HF, McKeague IW (2006). The development of the sexual self‐concept inventory for early adolescent girls. Psychol. Women Q.

[10] O’Sullivan LF, Brooks-Gunn J (2005). The timing of changes in girls’ sexual cognitions and behaviors in early adolescence: A prospective, cohort study. J. Adolesc. Health.

[11] Valencia CP, Canaval GE, Sevilla TM, Orcasita LT (2015). Sexual debut in young adults in Cali as transition: keys for care. Invest. Educ. Enferm.

[12] Pai HC, Lee S, Yen WJ (2012). The effect of sexual self‐concept on sexual health behavioural intentions: a test of moderating mechanisms in early adolescent girls. J. Adv. Nurs.

[13] Pai HC, Lee S (2012). Sexual self‐concept as influencing intended sexual health behaviour of young adolescent Taiwanese girls. J. Clin. Nurs.

[14] Parkes A, Henderson M, Wight D, Nixon C (2011). Is parenting associated with teenagers’ early sexual risk‐taking, autonomy and relationship with sexual partners?.. Perspect. Sex. Reprod. Health.

[15] Sánchez R, Echeverry J (2004). Validación de escalas de medición en salud. Revista de Salud pública.

[16] Alberto Arribas (2006). Adaptación transcultural de instrumentos. Guía para el proceso de validación de instrumentos de tipo encuestas. Rev. Asoc. Med Bahía Blanca..

[17] Urrutia-Egaña M, Barrios-Araya S, Núñez MG, Camus MM (2014). Métodos óptimos para determinar validez de contenido. Rev. Cub. Educ.Méd. Super.

[18] Tristán- López A (2008). Modificación al modelo de Lawshe para el dictamen cuantitativo de la validez de contenido de un instrumento objetivo. Avances en medición.

[19] Herrera BS, González GM, Cardenas DC, Alarcón AA (2017). Diseño, validez facial y de contenido del instrumento carga de la enfermedad crónica para el paciente-GPCP-UN. Rev. Méd Risaralda.

[20] Fleiss JL (1999). The design and analysis of clinical experiments.

[21] Hutchinson A, Bentzen N, Konig-Zanhn C (1997). Cross cultural health outcome assessment: a user’s guide.

[22] Rincón-Álvarez DA, Chaparro-Díaz L (2017). Validity and Reliability of the Spanish Version of the Technological Competency as Caring in Nursing Instrument. Invest. Educ. Enferm.

[23] González GMC, Herrara BS, Rosero EV (2016). Desarrollo y pruebas psicométricas del Instrumento "cuidar"-versión corta para medir la competencia de cuidado en el hogar. Rev. Salud UIS.

[24] Vesga-Gualdrón LM, Ruiz CH (2016). Validez y confiabilidad de una escala de cuidado profesional en español. Av. Enferm.

[25] Galeano EMM, Cuevas VMC (2016). Validez y confiabilidad del instrumento "Medición de la autoeficacia percibida en apnea del sueño"-SEMSA. Versión en español. Aquichan.

[26] Polit DF, Hungler BP (2005). Investigación Científica en Ciencias de la Salud.

[27] Morales MNP, Ruiz CH (2013). Adecuación semántica de la Escala de Cuidado Profesional (CPS).. Aquichan.

